# Three-Dimensional Silk Fibroin/Chitosan Based Microscaffold for Anticancer Drug Screening

**DOI:** 10.3389/fbioe.2022.800830

**Published:** 2022-03-08

**Authors:** Hui Niu, Jiarui Xiao, Xiaoli Lou, Lingling Guo, Yongsheng Zhang, Runhuai Yang, Hao Yang, Shouli Wang, Fuzhou Niu

**Affiliations:** ^1^ Department of Pathology, Second Affiliated Hospital of Soochow University, Suzhou, China; ^2^ School of Biology and Basic Medical Sciences, Soochow University, Suzhou, China; ^3^ School of Life Science, Anhui Medical University, Hefei, China; ^4^ Robotics and Microsystems Center, College of Mechanical and Electrical Engineering, Soochow University, Suzhou, China; ^5^ School of Mechanical Engineering, Suzhou University of Science and Technology, Suzhou, China

**Keywords:** microenvironment, biomaterials, drug screening, silk fibroin/chitosan scaffold, *in vitro* tumor research model

## Abstract

Traditional monolayer cell cultures often fail to accurately predict the anticancer activity of drug candidates, as they do not recapitulate the natural microenvironment. Recently, three-dimensional (3D) culture systems have been increasingly applied to cancer research and drug screening. Materials with good biocompatibility are crucial to create a 3D tumor microenvironment involved in such systems. In this study, natural silk fibroin (SF) and chitosan (CS) were selected as the raw materials to fabricate 3D microscaffolds; Besides, sodium tripolyphosphate (TPP), and 1-ethyl-3-(3-dimethylaminopropyl)-carbodiimide (EDC) were used as cross-linking agents. The physicochemical properties of obtained scaffolds were characterized with kinds of testing methods, including emission scanning electron microscopy, x-ray photoelectron spectroscopy, fourier transform infrared spectroscopy, water absorption, and swelling ratio analysis. Cancer cell lines (LoVo and MDA-MB-231) were then seeded on scaffolds for biocompatibility examination and drug sensitivity tests. SEM results showed that EDC cross-linked scaffolds had smaller and more uniform pores with great interconnection than the TPP cross-linked scaffolds, and the EDC cross-linked scaffold exhibited a water absorption ratio around 1000% and a swelling ratio of about 72%. These spatial structures and physical properties could provide more adhesion sites and sufficient nutrients for cell growth. Moreover, both LoVo and MDA-MB-231 cells cultured on the EDC cross-linked scaffold exhibited good adhesion and spreading. CCK8 results showed that increased chemotherapeutic drug sensitivity was observed in 3D culture compared with 2D culture, particularly in the condition of low drug dose (<1 μ M). The proposed SF/CS microscaffold can provide a promising *in vitro* platform for the efficacy prediction and sensitivity screening of anticancer drugs.

## Introduction

Most anticancer drugs that show promise in preclinical studies exhibit less or no benefit in later clinical trials, and only less than 5% of new anticancer drugs were approved (Sant and Johnston, 2017). One major cause of such a high failure rate is that conventional preclinical models can’t accurately predict the efficacy and toxicity of drug candidates (Dhandapani and Goldman, 2017). Anticancer drug screening can be achieved using *in vivo* and *in vitro* methods. The application of *in vivo* models, such as patient-derived xenografts (PDX), is limited by their complexity, high cost, and associated ethical issues (Murayama and Gotoh, 2019; Invrea et al., 2020). The majority of *in vitro* assays are based on traditional two-dimensional (2D) cultures of cancer cell lines, where cells are grown on a flat surface and/or form a monolayer. However, the 2D monolayer can’t effectively mimic the natural tumor microenvironment (Fontoura et al., 2020). Recently, various 3D culture models have been studied to recapitulate the tumor microenvironment (Agarwal et al., 2017; Gomez-Roman et al., 2017; Rijal and Li, 2017; Lee et al., 2018). At present, three-dimensional models include vivo-like models, microarray technology; tumor-on-a-chip platforms, pre-fabricated engineered scaffolds, scaffolds-free, liquid-overlay culture, gyratory rotation, and spinner flask spheroid cultures, and so on (Benton et al., 2014; Negrei et al., 2016; Franchi-Mendes et al., 2021; Liu et al., 2021). It has been found that cancer cells grown in 3D culture systems display different morphological and physiological properties from those in 2D culture. Overall, 3D cancer models are better to represent *in vivo* tissue and predict drug response than 2D culture systems (Kapałczyńska et al., 2018; Lim and Park, 2018).

3D scaffolding is one of the most common 3D culture techniques that has been increasingly used in tissue engineering, cancer research, and drug delivery (Shakibaei et al., 2015; Roseti et al., 2017; Wani and Veerabhadrappa, 2018; Limongi et al., 2020; Yang et al., 2020). The selection of biomaterials plays a critical role in determining the properties of obtained scaffolds and the quality of subsequent cell culture and data interpretation. Silk fibroin (SF) is a natural fibrous protein characterized by high oxygen and water permeability, good biocompatibility, and robust mechanical strength (Huang et al., 2018). SF-based nanoparticles were investigated to be used as carriers in a novel drug-delivery system, which showed a good encapsulation efficiency and release profile (Hudita et al., 2021; Radu et al., 2021). SF-based scaffolds prepared by different methods often have different biological characteristics. For instance, the porous scaffolds prepared by the salt leaching method usually have poor connectivity and cannot control the shape of the pores (Vishwanath et al., 2016). The gas foaming method avoids the use of chemical solvents and reduces the fabrication time. However, the disadvantage is that scaffolds prepared by this method usually have relatively small closed pores (Zeng et al., 2015). What’s exciting is that the freeze-drying method has good application value. In freeze-drying, the porous structure of scaffolds relies on the formation of ice crystals, which is largely dependent on the parameters of prepared solutions. Previous studies of SF-based scaffolds used different concentrations of SF solution ranging from 2 to 10% (Mukhopadhyay et al., 2017; Wang et al., 2020). More importantly, Li et al. reported that when SF/CS ratio was 1:1, the porosity and the water uptake ratio of obtained scaffolds significantly decreased as the SF/CS concentration increased from 2 to 12% (Li et al., 2017b). Thus, although the standardized protocol of SF solution preparation has been described (Huang et al., 2018), there is no consensus on the method of scaffold construction. Moreover, using only SF material to fabricate scaffolds may lead to insufficient stability in water and poor cell adhesion (Fan et al., 2020; Luetchford et al., 2020). Improved properties of SF-based scaffolds can be achieved by blending with other polymers, such as Chitosan (CS). However, the technical parameters for SF/CS microscaffold fabrication process need to be better stated; And, the effects of cross-linking agents affiliated with the scaffolds should be estimated. Besides, the application of using SF/CS microscaffolds for drug sensitivity screening was few reported.

In this study, we prepared SF/CS composite scaffolds with two kinds of cross-linking agents, sodium tripolyphosphate (TPP) and1-ethyl-3-(3-dimethylaminopropyl)-carbodiimide (EDC). The properties of scaffolds were characterized using scanning electron microscopy (SEM), attenuated total reflectance fourier transform infrared spectroscopy (ATR-FTIR), X-ray diffraction analysis (XRD), water absorption analysis, and swelling ratio analysis. LoVo cells were seeded on the scaffolds to observe cell growth and adhesion, and cell proliferation was examined with MTT assay. Finally, Lovo cells and MDA-MB-231 were used to test the chemotherapeutic drug sensitivity in three culture environments: traditional 2D culture, 3D SF/CS scaffold, and 3D SF/CS scaffold containing extract of tumor tissue. Results showed that 3D SF/CS scaffold cross-linked by EDC provides a suitable environment for cancer cell growth and has potential applications in cancer research.

## Materials and Methods

### Cells and Reagents

LoVo human colon cancer and MDA-MB-231 human breast cancer cell lines were obtained from American Type Culture Collection (United States of America). 5-fluorouracil (5-FU), methotrexate (MTX), paclitaxel (PTX), oxaliplatin (OXA), irinotecan (CPT-11), and capecitabine were purchased from Sinopharm Chemical Reagent (China). CS was purchased from AK Biotech Co., Ltd. (China). Dulbecco’s modified Eagle’s medium (DMEM), fetal bovine serum (FBS), and phosphate-buffered saline (PBS) were from Gibco (USA). TPP was obtained from Invitrogen (USA). EDC, N-hydroxysuccinimide (NHS), lithium bromide, 1% penicillin-streptomycin, 3-(4,5-dimethylthiazol-2-yl)-2,5-diphenyltetrazoliumbromide (MTT), and dimethyl sulfoxide (DMSO) were purchased from Sigma-Aldrich (USA). Glacial acetic acid was obtained from Sinopharm Chemical Reagent (China). Cell counting kit-8 (CCK-8) reagent was from Beyotime Biotechnology (China).

### Preparation of SF Solution

The preparation of the SF solution followed the procedures described previously (Huang et al., 2018). *Bombyx mori* cocoons (Maoda Textile, China) were cut into small pieces and boiled in 0.5% sodium carbonate solutions for 3 h. The shells were washed twice with double distilled water and placed in a 60°C oven until the weight did not change. Dried shells were then dissolved in 9.3M lithium bromide solution and underwent dialysis to obtain an aqueous SF solution. The final concentration of SF solution was approximately 3% w/v.

### Preparation of Chitosan Solution

At room temperature, 3 g chitosan (Aokang Biotechnology, China) was dissolved in 100 ml of glacial acetic acid solution with a concentration of 0.1 mol/L (pH = 4.6). The mixed solution was stirred thoroughly until it became clear to obtain 3% (w/v) chitosan solution.

### Fabrication Process of SF/CS Composite Microscaffolds

The SF and CS solutions were first mixed in 1:1 proportion and then cross-linked by the addition of 50 mmol/L EDC and 18 mmol/L NHS or 1 mg/ml TPP solution. The mixture was processed with a gradient freezing technique followed by drying in a vacuum freeze dryer for 36–48 h to obtain EDC cross-linked and TPP-linked SF/CS scaffolds. To improve water stability, the dry prepared scaffolds were then immersed in anhydrous methanol and 10% sodium hydroxide solutions (1:1 proportion) (Tong et al., 2015; Zeng et al., 2015; Ruan et al., 2017), washed three times with deionized water, and dried in the freeze dryer for 36–48 h. SF and CS solutions were processed in the same manner to obtain pure SF and CS scaffolds.

### Scanning Electron Microscopy

The microstructures of the scaffolds were detected through SEM with a JEOL JSM6460LV microscope (S-4700; Hitachi, USA). The samples were coated with sputtered gold, and their outer and inner sections were prepared by breaking scaffolds in liquid nitrogen.

### Attenuated Total Reflectance Fourier Transform Infrared Spectroscopy

Measurement of the scaffolds structure ware carried out with infrared spectroscopy with an ATR-FTIR spectrophotometer (NICOLET 560, USA). The resolution was 4 cm^−1^ after the accumulation of 256 scans for each spectrum. The scanning range was 2,000–400 cm^−1^.

### X-Ray Diffraction Analysis

The microstructures of the crystalline and amorphous materials in scaffolds were studied with XRD with a fully automated X-ray diffractometer (Bruker, Germany). The experimental parameters were as follows: copper target, LynxExe array detector, 40 kV, 40 mA, scanning step length of 0.04°, and scanning speed of 35.4 s/step.

### Analysis of Water Absorption and Swelling Ratio

Scaffolds of certain weights were soaked in double-distilled water for 24 h. After the water was removed from the surface, the weights of the wet scaffolds were recorded as M1. The scaffolds were then dried at 60°C until the weight did not change, and the weight was recorded as M2.

$$\mathrm{The}\;\mathrm{rate}\;\mathrm{of}\;\mathrm{waterabsorption}=\frac{M_{2}-M_{1}}{M_{2}}\times 100\%$$ (1)

Certain volumes of scaffolds were used and measured as V1. The scaffolds were then soaked in double-distilled water for 24 h. After the water was removed from the surface, the volume was measured as V2.

$$\mathrm{The}\;\mathrm{swelling}\;\mathrm{ratio}=\frac{V_{2}-V_{1}}{V_{1}}\times 100\%$$ (2)

### Cell Culture

For 2D culture, Lovo cells were maintained in DMEM containing 10% FBS and 1% penicillin-streptomycin. All cells were cultured at 37°C in a humidified atmosphere containing 5% CO2. For 3D culture, 3D scaffold samples were cut into circular discs for 96-well plates (Corning, USA) and sterilized under ultraviolet light. The circular matrices were immersed in 75% alcohol three times before they were used. Cells were suspended at the proper density on scaffolds and then rinsed extensively three times with sterile PBS and kept in DMEM medium for 1 h. Cells were then maintained in DMEM and incubated at 37°C, as with 2D culture.

### Preparation of Fresh Tumor Tissue Extract

All procedures using mice were reviewed and approved by the Ethic Committee of Soochow University, implemented according to institutional animal ethics guidelines for the Care and Use of Research Animals established by Soochow University, and reported in adherence to the ARRIVE guidelines (Percie du Sert et al., 2020). Cancer cell (Lovo cells and MDA-MB-231cells) suspensions (1 × 10^6^ cells/mouse) were subcutaneously injected into ten BALB/c nude mice (5–6 weeks old, 18 ± 2 g on average). Subcutaneous tumor tissue was obtained when the tumor size was around 10 mm × 10 mm, and then placed in a tissue homogenizer with liquid nitrogen for rapid grinding. The tissue was then homogenized in sterile PBS solution and centrifuged for 25 min (4°C, 15,000 rpm). The supernatant was collected and filtered with a 0.22 µm filter to remove bacteria. The filtered extract was then stored in a cryopreservation tube at -80°C. The total protein concentration of the tumor tissue extract was calculated with a UV/Vis spectrophotometer.

### MTT Assay

A total of 5,000 cells/well (100 μ l, 5 × 10^4^cells/ml) were placed in 96-well plates in DMEM containing 10% FBS for 24 h. The cells were then incubated with 20 μl MTT (5 mg/ml) solution for 4 h. The MTT solution was removed, and the cells were incubated with 150 μL DMSO. A microplate reader (Thermo Scientific, USA) was used to measure the optical density at 560 nm.

### Culture Model and Drugs Treatments

Cells were cultured in 2D plates and 3D plates as follows (determined on the basis of preliminary experiments): LoVo, 5,000 cells/well (2D) and 10,000 cells/well (3D); MDA-MB-231, 5,000 cells/well (2D) and 10,000 cells/well (3D). The drugs were added to the 2D plates after 24 h and to the 3D plates after 5 days. These drugs are 5-fluorouracil (5-FU), methotrexate (MTX), paclitaxel (PTX), oxaliplatin (OXA), irinotecan (CPT-11), and capecitabine with different concentration gradients (0.01, 0.1, 1, 10, and 100 μ M), which are purchased from Sinopharm Chemical Reagent (China). The sensitivity of cells to chemotherapeutic drugs was measured by CCK8 after 48 h of drugs treatment. Experimental control wells contained only cancer cells without drugs; experimental wells contained both cells and drugs; and blank control wells contained no cells or drugs.

### CCK-8 Assay

Cell viability was accessed by CCK-8 assay after 48 h of drug treatment. Cells were incubated in 20 μL CCK-8 solution at 37°C for 2 h. A microplate reader (Thermo Fisher Scientific, USA) was used to measure the optical density (OD) at 450 nm. CCK-8 assays were replicated three times for each formulation and culture period. The inhibition ratio (IR) of chemotherapeutic drugs.

$$\mathrm{IR}=\frac{OD_{\exp\mathrm{erimental}\;\;\mathrm{control}}\mathrm{-O}D_{\exp\mathrm{erimental}}}{OD_{\exp\mathrm{erimental}\;\mathrm{control}}\mathrm{-OD}{\;}_{\mathrm{blank}\;\mathrm{control}}}\times 100\%$$ (3)

### Statistical Analysis

All experiments were performed in triplicate and at least three times. Data were expressed as mean ± standard deviation. The student’s two-tailed *t*-test was performed with GraphPad Prism v.6 software (GraphPad Software, Inc., San Diego, CA) to compare the differences between treated groups and the corresponding control groups. A *p*-value <0.05 was considered statistically significant.

## Results

### Effects of Different Cross-Linking Agents on 3D Scaffold Morphology

**FIGURE 1:**
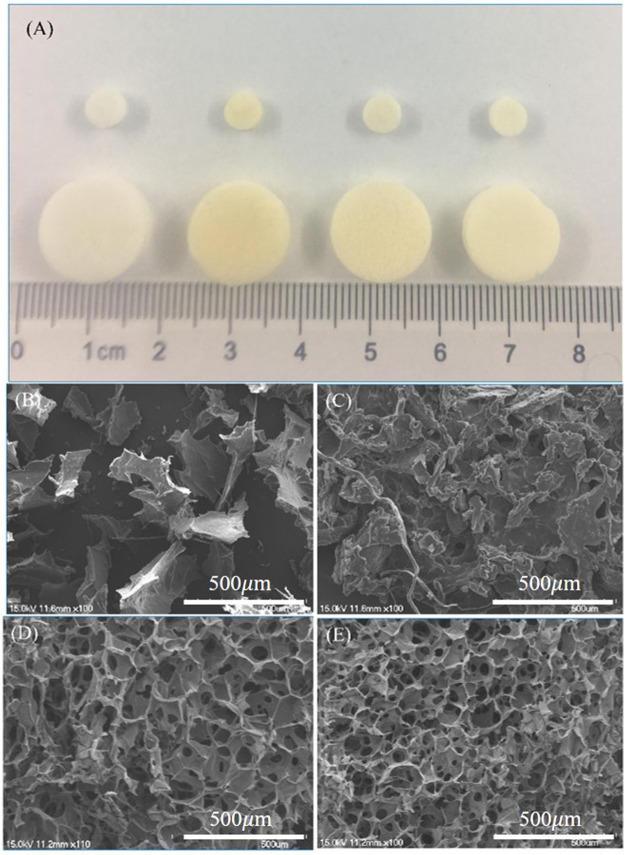
Macroscopic and microscopic views of different 3D scaffolds. **(A)** The gross morphology of 3D scaffolds. SEM micrographs of scaffolds made of **(B)** pure SF, **(C)** pure CS, **(D)** SF/CS cross-linked by TPP, and **(E)** SF/CS cross-linked by EDC.

Each group of scaffolds was prepared with 24-well and 96-well plates. From left to right, the scaffolds were pure SF scaffold, pure CS scaffold, TPP cross-linked SF/CS scaffold and EDC cross-linked SF/CS scaffold (Figure 1A). The pure SF scaffold was white and brittle. The pure CS scaffold was yellow and soft. TPP and EDC cross-linked SF/CS scaffolds were yellowish-white with rough surfaces and a sponge-like texture.

To observe the internal structures of the scaffolds, we examined each group with SEM (Figures 1B–E). The pure SF scaffold had an unstable, coiled, and flaky structure. The pure CS scaffold formed irregular spaces with poor interconnection, which were unfavorable to the transport of nutrients. Both TPP and EDC cross-linked SF/CS scaffolds formed regular, small pore structures. Compared with TPP scaffolds, EDC cross-linked scaffolds had smaller and more uniform pores with great interconnection.

### Physicochemical Properties of SF/CS Scaffolds With Different Cross-Linking Agents

#### ATR-FTIR

**FIGURE 2:**
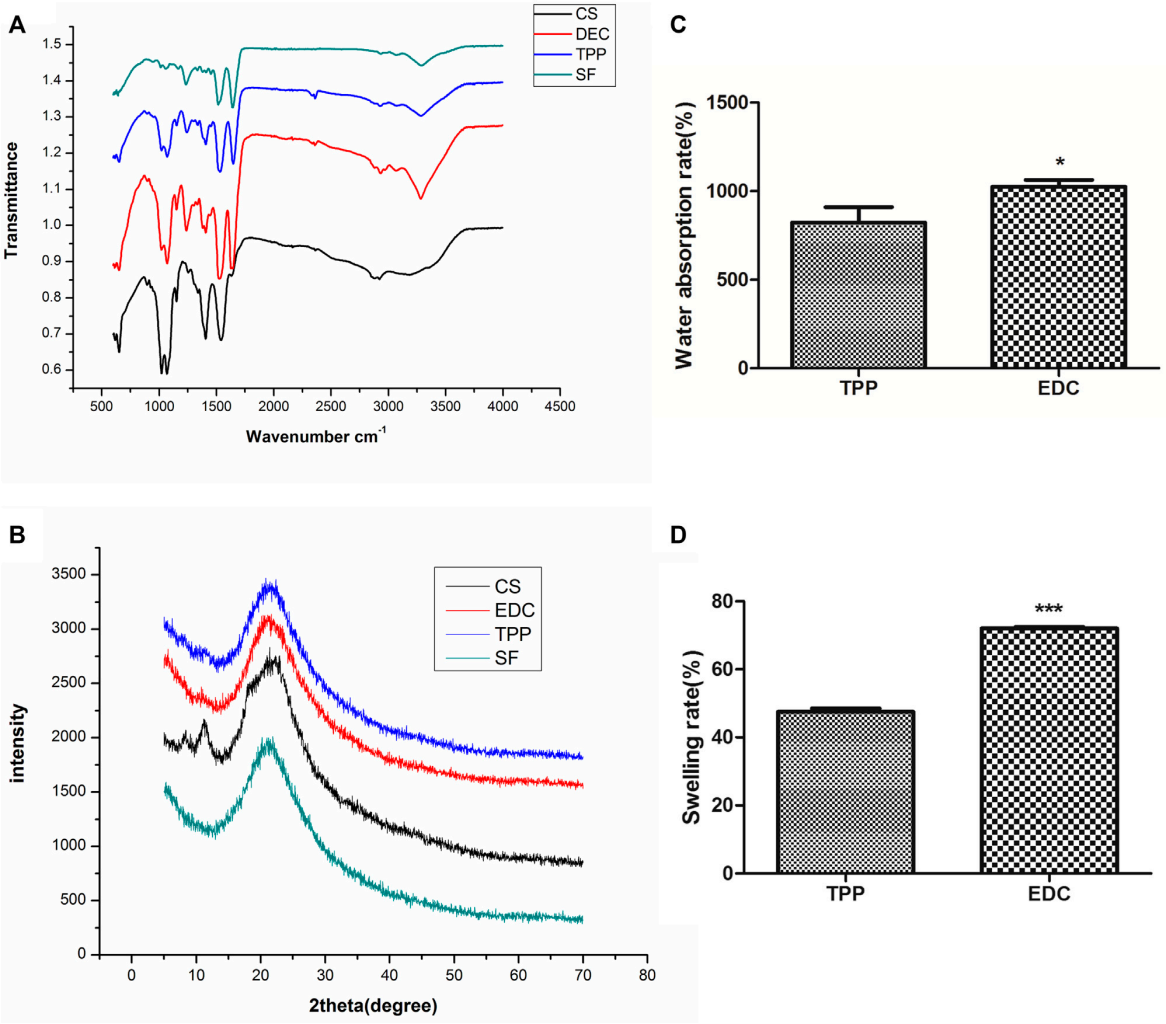
Physicochemical properties of different 3D scaffolds. **(A)** ATR-FTIR. **(B)** XRD spectra. **(C)** Water absorption rate. **(D)** Swelling ratio.

The functional group composition in the four groups of scaffolds is shown in the ATR-FTIR spectra (Figure 2A). The characteristic peaks at 1,625 cm^−1^, 1,529 cm^−1^, and 1,236 cm^−1^ represented the amide I, II, and III of SF, respectively. Pure CS alone had a characteristic absorption peak at 1,035–1,154 cm^−1^, and its NH2 characteristic peak appeared at 1,558 cm^−1^ because its β-(1,4) glycosidic bond was interconnected. Its amide I band at 1,623 cm^−1^ was weak, because the CS used in the experiment had a 90% deacetylation. The amide I band of pure SF was between 1,650 and 1,660 cm^−1^ (1,654 cm^−1^), thus indicating that SF has an α helix or an irregular crimped structure. The spectra of SF/CS scaffolds cross-linked by EDC or TPP showed amide I bands between 1,625 and 1,634 cm^−1^, representing a more stable β-folded structure than that of pure SF and pure CS.

#### XRD

The analysis of basic composition in the four groups of scaffolds is shown in the XRD spectra (Figure 2B). The pure CS scaffold had the main peak at 2θ = 20.1° that corresponded to the low crystallization of CS. The 2θ peak of the pure SF scaffold was broad and appeared at 20.9°, which was characteristic of amorphous SF (α-helix or random coil structure). The peaks of EDC and TPP cross-linked SF/CS scaffolds appeared at 21.7° and 21.4° respectively, indicating that the process of cross-linking increased the crystallinity of individual materials.

#### Analysis of Water Absorption Rate and Swelling Ratio

In this study, only cross-linked SF/CS scaffolds were tested because the pure SF group and pure CS group were unstable and soluble in water. The results are shown in Figures 2C,D the EDC group had a rate of water absorption of 1000% or greater, whereas that of the TPP group was only approximately 800%. The swelling ratio of the EDC group was 72% and that of the TPP group was only approximately 47%.

### Cell Proliferation in SF/CS Scaffolds Constructed by Different Cross-Linking Agents

#### MTT

**FIGURE 3:**
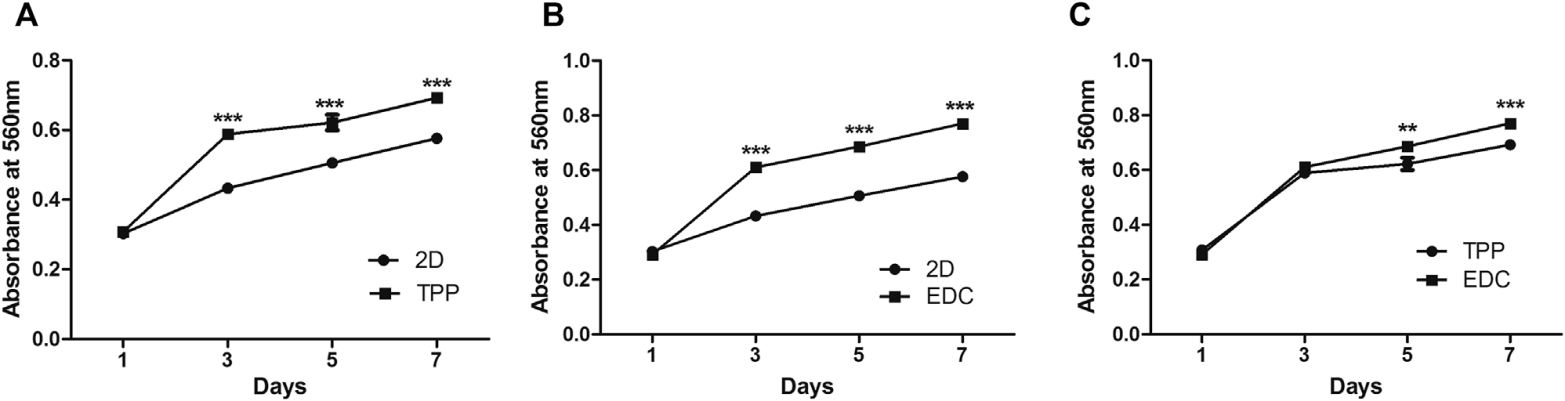
Comparison of LoVo cell proliferation by MTT assay between two of the three conditions: namely a 2D environment, TPP cross-linked SF/CS scaffold, and EDC cross-linked SF/CS scaffold: **(A)** 2D vs TPP. **(B)** 2D vs EDC. **(C)** TPP vs EDC. **p < 0.01, ***p < 0.001.

The proliferation of cancer cells in 2D and 3D (TPP and EDC groups) environments were examined with MTT assays after 1, 3, 5, and 7 days of culture (Figure 3). On the first day, cancer cells mainly began early adhesion after being seeded on the scaffold material. On the third day, cells began to undergo rapid proliferation. On the fifth day, cells continued to proliferate, and the difference between the control group and the experimental group was statistically significant (*p* < 0.001), thus indicating that the cells grew better in 3D culture (Figures 3A,B). The difference in the proliferation rate between the two 3D groups was also significant (*p* < 0.001) (Figure 3C). The cells in the EDC group displayed superior proliferation ability to that of the TPP group.

#### SEM

**FIGURE 4:**
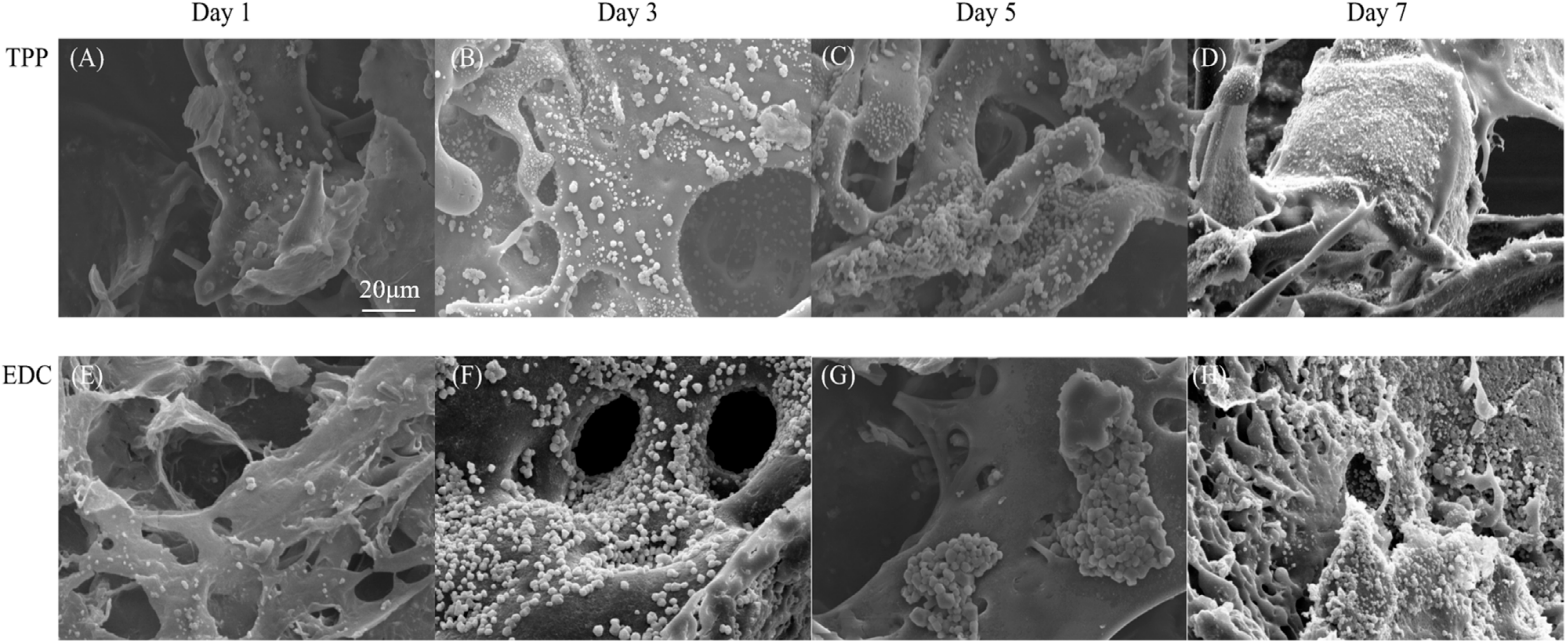
SEM micrographs of LoVo cells cultivated on **(A-D)** TPP cross-linked scaffolds and **(E-H)** EDC cross-linked scaffolds after 1 day, 3 days, 5 days, and 7 days.

The morphology of cancer cells in the TPP group and the EDC group were observed under SEM (Figure 4). In the TPP group, cancer cells were first scattered on the surfaces of the scaffolds (Figure 4A). After 3 days, small amounts of granular substances appeared around the cells (Figure 4B). After 5 days, large numbers of cells formed lump-like structures in the scaffold pores. The cell proliferation was active, and the amount of granular material around the cells increased (Figure 4C). The appearance of granular substances may be due to the degradation of dead cells, extracellular matrix, and scaffold material. The degradation of scaffold pore structures made the pore wall thinner or even caused it to disappear (Figure 4D). Compared with the TPP group, the EDC group exhibited better cell compatibility. The cells adhered to the scaffold and formed antenna-like structures conducive to cell division and proliferation (Figures 4E,F). After 5 days, the cells grew well, and the agglomerate grew towards the surroundings. The growth pattern was close to the infiltration pattern of cancer cells *in vivo* (Figures 4G,H). Interestingly, pore structures with different sizes formed on the surface of the cell lump, which may facilitate the transport of nutrients and metabolic substrates. The formation of pore structures in the scaffold was comparable to the process of tumor vascularization *in vivo*.

### Effects of Tumor Tissue Extract on Cell Proliferation in EDC Cross-Linked SF/CS Scaffold

**FIGURE 5:**
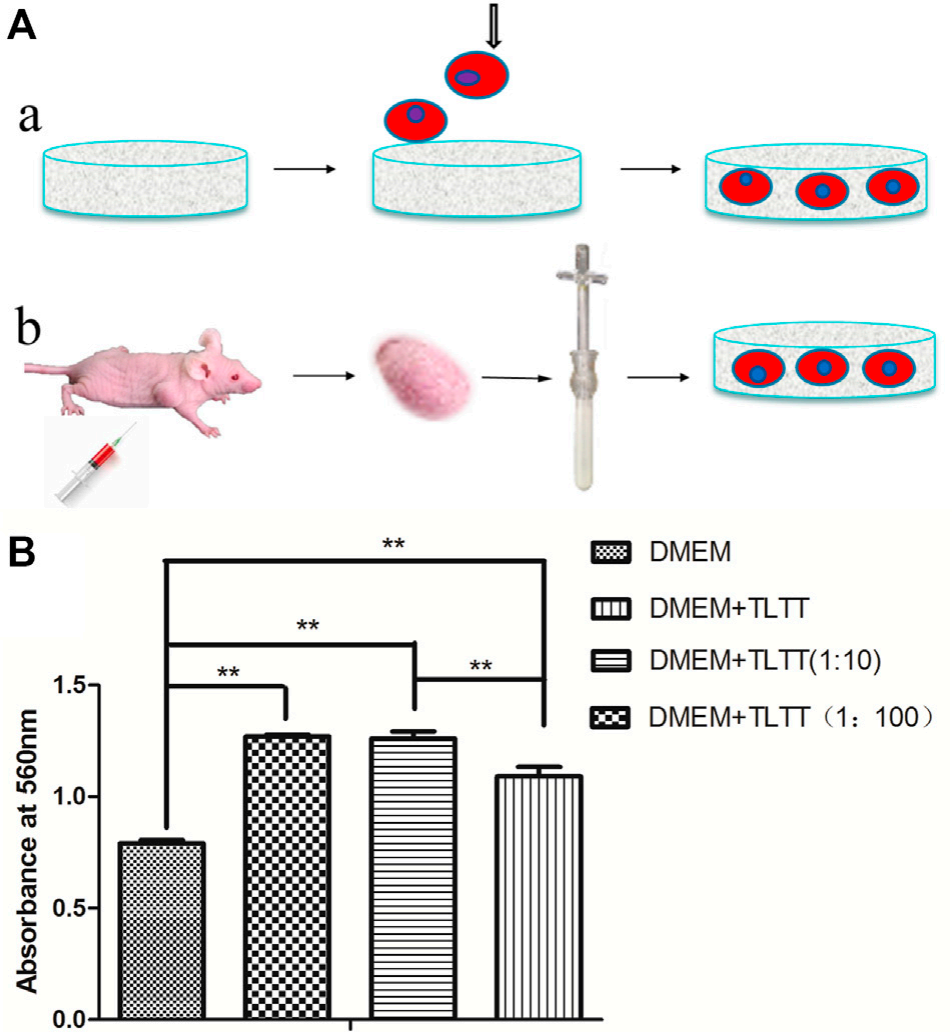
**(A)** Schematic diagram of 3D tumor microenvironment by growing cancer cells on porous scaffolds **(A)** and adding tumor tissue extract to the culture system **(B)**. **(B)** Effects of tumor tissue extract with different proportions on cancer cell proliferation. **p < 0.01, (DMEM: Dulbecco’s Modified Eagle Medium containing 2% fetal bovine serum; TLTT: Stock solution of tumor tissue extract, TLTT (1:10): 10-fold diluted tumor tissue extract; TLTT (1:100): 100-fold diluted tumor tissue extract).

To simulate the tumor microenvironment *in vitro*, we added tumor tissue extract to 3D cell culture to provide the cytokines and signal transduction molecules needed for cell growth (Figures 5A,B). The effects of different proportions of tumor tissue extract on cell proliferation were detected with MTT assays (Figure 5B). The difference was statistically significant (*p* < 0.05) with 10-fold and 100-fold diluted tumor tissue fluid extract. Tumor tissue extract was beneficial to the proliferation of cancer cells under certain conditions.

### *In Vitro* Evaluation of Chemosensitivity

Based on the test results of physicochemical properties and cell compatibility, we used EDC cross-linked SF/CS scaffold for the initial testing of chemotherapeutic drug sensitivity. Five different concentrations of chemotherapeutic drugs (namely 0.01, 0.1, 1, 10, and 100 µM) were added to the traditional 2D culture (2D group), a simple SF/CS scaffold (3D group), and an SF/CS scaffold with fresh tumor tissue extract (3D + TLTT group). The sensitivity of cells to chemotherapeutic drugs was measured by CCK8 after 48 h of drugs treatment. The results were interpreted as resistant (IR<30%), moderately sensitive (30% ≤ IR ≤ 50%), or sensitive (IR>50%).

**FIGURE 6:**
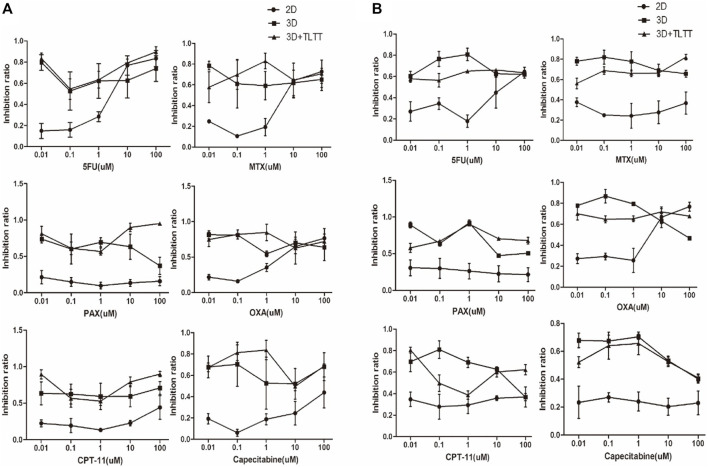
The chemosensitivity of **(A)** LoVo cells and **(B)** MDA-MB-231 cells in 2D, 3D, and 3D + TLTT culture environments.

Figure 6 illustrates the chemosensitivity of both LoVo cells and MDA-MB-231 cells in 2D, 3D, and 3D + TLTT culture environments. It can be found that the chemosensitivity in 2D and 3D culture significantly differed and higher sensitivity was observed in the 3D environment when the drug concentrations were low. No significant differences were seen between the two types of 3D environment (3D and 3D + TLTT), thus indicating that the addition of tumor tissue fluid extract did not significantly affect the sensitivity to chemotherapeutic drugs. The IR data of Figure 6 are provided in Supplementary Tables S1, S2 respectively.

## Discussion

With the development of 3D culture technology, people have a new understanding and development of tumor model. The construction and application of 3D tumor model will become an inevitable trend. To date, studies showed SF is of good biocompatibility, slow degradation rate, low immunogenicity (Gholipourmalekabadi et al., 2020; Guan et al., 2020; Chen et al., 2021). And, SF-based scaffolds have been applied in diverse studies of *in vitro* tumor models (Talukdar et al., 2011; Mishra et al., 2019). SF/CS scaffolds could provide not only the space for cell tissue to form a three-dimensional (3D) structure, but also the mechanical integrity and hydration space for the diffusion of nutrients and metabolites in cells (Guan et al., 2013; Li et al., 2017b).

In the study, we established a 3D SF/CS composite scaffold that could be potentially used for the pre-screening of chemotherapeutic drugs. As high porosity and water uptake ratio are desirable for cell growth and material exchange, we selected the relatively low total concentration for scaffold fabrication in our study. The pure SF scaffold is brittle and unstable in water. The physical properties of SF scaffolds can be improved by mixing with other synthetic or natural polymers (Gobin et al., 2005; Lv and Feng, 2006). Blending SF with CS is an interaction of hydrogen bonding, causing the formation of a stable β-sheet conformation in SF (Tian et al., 2017). Usually, different cross-linking agents are used in different culture models. Cross-linking agents can be added to further stabilize the structure. At present, the crosslinking agents used in the research include EDC/NHS, TPP, glutaraldehyde (GA) and so on (Teimouri et al., 2015; Ruan et al., 2017; Auwal et al., 2018). GA crosslink can improve considerably the molecular stability and antidegradation of the chitosan (CS) solution. However, GA has certain cytotoxicity and is generally used for tissue fixation. Therefore, it is not the preferred material for our cell culture scaffolds. EDC/NHS is an alternative crosslinking agent for GA. it can not only improve the mechanical properties of scaffolds, but also rarely have cytotoxic reactions, and has good biocompatibility (Lehmann et al., 2017). In the process of preparing stratified collagen/CH-PCL scaffolds, TPP can more effectively crosslink with the amino group of chitosan (Zhu et al., 2014). Inspired by those previous works, we prepared SF/CS scaffolds with two different crosslinking agents (EDC or TPP), and compared their morphological and physicochemical properties.

In order to explore whether different biological crosslinking agents have different effects on SF/CS 3D scaffolds. Firstly, we observed the external morphology and internal structure of the 3D scaffolds by ordinary light microscope and SEM. The experimental results show that the unstable crimp structure and lamellar structure of silk fibroin can form porous SF/CS 3D scaffolds with chitosan solution under the action of biological crosslinking agent. The two biological crosslinking agents play different roles in the formation of voids in SF/CS 3D scaffolds, and the voids formed by EDC crosslinked SF/CS 3D scaffolds are more uniform and better connected than TPP crosslinked SF/CS 3D scaffolds. In addition, ART-FTIR and XRD showed that no new chemical bond was formed in the composite process, but a simple physical bond. Moreover, the degree of crystallization peak of cross-linked scaffolds is not lower than that of pure chitosan scaffolds, which may be because the cross-linking agent increases the degree of crystallization of mixed scaffolds. Besides, compared with TPP group, EDC crosslinked SF/CS 3D scaffolds has moderate water absorption (1000%) and swelling (72%), which may provide sufficient nutrients for the growth process of cells. Most importantly, cell proliferation experiments (MTT and SEM) confirmed that EDC scaffolds were more conducive to cell growth. Through comparison and physicochemical properties, we conclude that EDC crosslinked SF/CS scaffolds could be used to obtain a good biocompatibility and structures to establish an *in vitro* tumor model, which was consistent with the results of Li et al. (Li et al., 2017a) and Zeng et al. (Zeng et al., 2015).

3D culture models have been found to profoundly affect cell growth and drug responses compared with traditional 2D culture. Our results showed that similar to the phenotypes displayed in other *in vitro* 3D tumor model, cancer cells seeded in SF/CS scaffolds grew in clusters and exhibited good adhesion (Liu et al., 2021). MTT assay showed the proliferation rate of cancer cells grown in SF/CS scaffolds was significantly higher than in 2D cell culture. Our present study tested the *in vitro* sensitivity of six chemotherapeutic drugs (5-FU, MTX, PTX, OXA, CPT-11, and capecitabine) on the LoVo and MDA-MB-231 cells. Compared with traditional 2D culture, the chemotherapeutic drug sensitivity was greater in 3D scaffolds, especially when the drug dose was low. This result is consistent with the previously reported drug sensitization effect of 3D cell culture (Shin et al., 2019). However, some studies have reported that tumor cells in 3D culture have higher drug resistance compared with traditional 2D culture (Fontoura et al., 2020). Hongisto et al. suggested that general conclusions cannot be drawn based on the observation of a single drug (Hongisto et al., 2013). Currently, we only tested the loVo and MDA-MB-231 cells, while further improvements and tests still need to be done.

3D tumor model not only provides 3D space for tumor cells to grow, but also reproduces the real growth of tumor cells in the body. Simulation of the tumor microenvironment requires both a 3D spatial structure and molecular components that facilitate cell growth *in vivo* (Rijal and Li, 2018). Here we not only analyzed the growth patterns of cancer cells in the prepared 3D scaffolds, but also investigated the effects of different proportions of tumor tissue extract on cell proliferation. Our results demonstrated that tumor tissue extract diluted by 10 or 100 times could promote cancer cell growth. However, 3D scaffolds with tumor tissue fluid extract did not significantly affect the sensitivity of chemotherapeutic drugs, thus indicating that cell proliferation of tumor cells may not correlate with the sensitivity of chemotherapeutic drugs. This may be the result of a variety of factors, and the specific mechanism still needs to be further studied.

## Conclusion

In conclusion, we demonstrated that a 3D SF/CS microscaffold cross-linked by EDC provides a suitable environment for cancer cell growth and has potential applications in cancer research. *In vitro* chemotherapeutic drug screening showed greater sensitivity of the 3D scaffold than the traditional 2D environment, when drugs were present in low doses. The EDC cross-linked 3D SF/CS scaffold may provide a promising new platform for *in vitro* evaluation and development of anticancer drugs.

## Data Availability

The original contributions presented in the study are included in the article/Supplementary Material, further inquiries can be directed to the corresponding authors.
