# Supplementary material for: Three-Dimensional Silk Fibroin/Chitosan Based Microscaffold for Anticancer Drug Screening
Source: Front Bioeng Biotechnol. 2022 Mar 8;10:800830. doi: 10.3389/fbioe.2022.800830 (PMC8957943; doi:10.3389/fbioe.2022.800830)
Supplement: Supplementary file 1 [file DataSheet1.PDF]

Supplementary Material

1 Supplementary Tables

1.1 Supplementary Table 1

Table 1. Chemotherapeutic drug sensitivity results of LoVo cells in different growing environment

**Drug Name**	**Cell Culture**	**Inhibition Ratio (IR)%**				
		**Concentration (µM)**				
		**0.01**	**0.1**	**1**	**10**	**100**
Fluorouracil (5-Fu)	2D	14.93	15.95	28.44	76.84	83.35
	3D	79.69	52.64	62.15	62.60	73.88
	3D+TLTT	83.50	54.55	63.32	79.21	89.59
Methotrexate (MTX)	2D	24.76	10.52	19.24	64.06	72.67
	3D	78.50	61.02	59.19	61.83	65.20
	3D+TLTT	57.69	69.68	82.88	64.58	70.62
Paclitaxel (PAX)	2D	21.38	14.89	9.86	13.54	15.90
	3D	73.84	60.19	69.44	63.27	37.04
	3D+TLTT	81.21	61.14	56.68	89.85	95.22
Oxaliplatin (OXA)	2D	21.70	15.94	35.28	64.89	76.58
	3D	81.43	81.04	54.59	70.04	63.77
	3D+TLTT	74.85	81.92	84.74	62.75	71.89
Irinotecan (CPT-11)	2D	22.47	19.34	13.35	22.83	44.13
	3D	63.59	62.55	59.37	59.62	70.89
	3D+TLTT	89.50	56.72	52.82	79.12	89.94
Capecitabine	2D	19.30	6.18	18.77	24.48	43.85
	3D	67.79	70.37	52.65	52.05	68.10
	3D+TLTT	67.50	81.27	83.72	49.59	68.74

2D: Traditional two-dimensional; 3D: Three-dimensional environment.

3D+TLTT: Three-dimensional environment with fresh tumor tissue fluid.

1.2 Supplementary Table 2

Table 2. Chemotherapeutic drug sensitivity results of MDA-MB-231 cells in different growing environment.

**Drug Name**	**Cell Culture**	**Inhibition Ratio (IR)%**				
		**Concentration (μM)**				
		**0.01**	**0.1**	**1**	**10**	**100**
Fluorouracil (5-Fu)	2D	26.98	34.35	17.91	44.72	64.45
	3D	60.15	76.48	80.68	62.54	61.99
	3D+TLTT	57.75	56.22	65.07	66.04	63.43
Methotrexate (MTX)	2D	37.59	24.85	24.21	27.65	36.71
	3D	77.93	81.99	77.83	68.88	65.74
	3D+TLTT	56.02	68.76	66.02	66.22	81.98
Paclitaxel (PAX)	2D	30.65	29.94	26.28	22.51	21.52
	3D	89.09	63.98	91.98	47.33	50.51
	3D+TLTT	57.96	66.99	90.17	70.41	67.82
Oxaliplatin (OXA)	2D	27.19	29.32	25.57	66.73	76.77
	3D	77.90	86.75	79.45	63.07	46.83
	3D+TLTT	70.07	64.76	65.28	71.78	67.80
Irinotecan (CPT-11)	2D	34.70	27.85	29.41	35.75	36.90
	3D	69.79	80.93	69.10	62.62	36.80
	3D+TLTT	80.52	49.69	38.71	60.09	62.24
Capecitabine	2D	23.39	27.03	24.05	20.34	22.98
	3D	67.79	67.34	70.38	53.26	40.15
	3D+TLTT	52.26	64.02	65.65	52.54	40.95

2D: Traditional two-dimensional; 3D: Three-dimensional environment.

3D+TLTT: Three-dimensional environment with fresh tumor tissue fluid.
